# Retraction: GDNF-Transfected Macrophages Produce Potent Neuroprotective Effects in Parkinson’s Disease Mouse Model

**DOI:** 10.1371/journal.pone.0314546

**Published:** 2024-12-17

**Authors:** The PLOS ONE Editors

Following publication of this article [1] an investigation by the University of North Carolina at Chapel Hill concluded that research misconduct occurred. Specifically, the investigation found falsification of research results as follows:

- In Fig 2, the β-actin and TSG101 loading controls are not from the same blots used to show GDNF expression.
- In Fig 5B, the western blot results shown for CD206 were obtained from unrelated lower molecular weight bands.

The *PLOS ONE* Editors note that image panels in Figs 7 and 8 of [1] appear similar to image panels in Figs 5 and 6 of [2] which was subsequently corrected in [3] to replace these figures. Additionally, in Fig 8 of [1], the lower left region of panel 8D appears similar to the central region of panel 8C; a replacement image was provided for panel 8C. The University of North Carolina at Chapel Hill investigated the issues with Figs 7 and 8 of [1], confirmed that the duplications between [1] and [2, 3] were due to errors in [2, 3], and did not find research misconduct occurred in these instances.

In light of the concerns about the reliability and integrity of the reported results in Figs 2 and 5 the *PLOS ONE* Editors retract this article.

The corresponding author EVB responded but did not confirm agreement nor disagreement with the editorial decision, and indicated that the wrong data were included in the article in error. EVB stands by the article’s findings and apologizes for the issues with the published article. YZ, MJH, RG, JPB, ZH, and AVK agreed with the retraction. JPB and AVK apologize for the issues with the published article.
